# Mind Bomb-1 in Dendritic Cells Is Specifically Required for Notch-mediated T Helper Type 2 Differentiation

**DOI:** 10.1371/journal.pone.0036359

**Published:** 2012-04-27

**Authors:** Hyun-Woo Jeong, Ji-Hoon Kim, Joo-Yeon Kim, Sang-Jun Ha, Young-Yun Kong

**Affiliations:** 1 Department of Biological Sciences, Seoul National University, Seoul, South Korea; 2 Department of Biochemistry, Yonsei University, Seoul, South Korea; International Center for Genetic Engineering and Biotechnology, India

## Abstract

In dendritic cell (DC)-CD4^+^ T cell interaction, Notch signaling has been implicated in the CD4^+^ T cell activation, proliferation, and subset differentiation. However, there has been a lot of debate on the exact role of Notch signaling. Here, we observed that expression of Mind bomb-1 (Mib1), a critical regulator of Notch ligands for the activation of Notch signaling, increases gradually as precursor cells differentiate into DCs in mice. To clarify the role of Mib1 in DC-CD4^+^ T cell interactions, we generated Mib1-null bone marrow–derived DCs. These cells readily expressed Notch ligands but failed to initiate Notch activation in the adjacent cells. Nevertheless, Mib1-null DCs were able to prime the activation and proliferation of CD4^+^ T cells, suggesting that Notch activation in CD4^+^ T cells is not required for these processes. Intriguingly, stimulation of CD4^+^ T cells with Mib1-null DCs resulted in dramatically diminished Th2 cell populations, while preserving Th1 cell populations, both in vitro and in vivo. Our results demonstrate that Mib1 in DCs is critical for the activation of Notch signaling in CD4^+^ T cells, and Notch signaling reinforces Th2 differentiation, but is not required for the activation or proliferation of the CD4^+^ T cells.

## Introduction

Dendritic cells (DCs) play a pivotal role as APCs in CD4^+^ T cell immune responses. Recently, Notch signaling has been implicated in DC-CD4^+^ T cell interaction, leading to activation, proliferation, and subset differentiation of CD4^+^ T cells. However, various experimental approaches have yielded disparate results and a conclusive consensus has not been reached yet [1], [2], [3]. Notch signaling is a highly conserved intercellular signaling pathway that regulates multiple cell-fate decisions. In mammals, Notch signaling is mediated by interactions between the four Notch receptors (Notch1-Notch4) and their ligands, Delta-like ligand-1 (Dll1), Dll3, Dll4, Jagged-1 (Jag1), and Jag2. The ligation to Notch receptors results in sequential proteolytic cleavages and release of the Notch intracellular domain (NICD), which translocates into the nucleus and functions as a transcriptional regulator for various target genes such as *Hes* and *Hey* [3], [4].

DCs and CD4^+^ T cells express Notch ligands, Dll1, Dll4, Jag1, and Jag2, and Notch receptors, Notch1 and Notch2, respectively, suggesting that Notch signaling is activated during DC-CD4^+^ T cell interaction [5], [6]. Several studies have reported that Notch signaling augments CD4^+^ T cell activation and proliferation [5], [7], [8], while other studies have suggested that Notch signaling negatively regulates T cell activation [9], [10]. There is more controversy on the role of Notch signaling with regard to Th differentiation. Many studies have reported that activation of Notch signaling promotes T-bet-mediated Th1 differentiation [7], [11], [12]. However, several other studies have suggested that Notch signaling is critical for Th2 differentiation but dispensable for Th1 differentiation [6], [13], [14]. Moreover, some studies have shown that Notch signaling cannot initiate Th1 or Th2 differentiation [15], [16]. These apparent inconsistencies among the many studies could be due to the nonphysiological practice of using chemicals and cytokines to stimulate or inhibit TCR and Notch signaling pathways [1], [2], [3]. In addition, the potential side effects of artificial APCs or of the direct manipulation of CD4^+^ T cells have not been well addressed. Thus, the exact role of Notch signaling in CD4^+^ T cells needs to be further determined by a novel genetic approach that minimizes the possible artifacts and side effects.

Recently, it was revealed that ligand internalization by endocytosis in the signal-sending cells is absolutely required for the initiation of Notch activation [17]. Four different E3 ubiquitin ligases, Mind bomb-1 (Mib1), Mib2, Neuralized-1 (Neur1), and Neur2 have been shown to regulate the endocytosis of Notch ligands in mice [18], [19], [20], [21], [22]; however, only Mib1 has been shown to play an obligatory role in the activation of Jag- as well as Dll-mediated Notch activation in vivo [23]. Therefore, cell-type-specific Mib1 conditional knockout mice have been known as excellent models for elucidating the role of Notch signaling in various contexts [24], [25], [26], [27], [28], [29].

To clarify the role of Notch signaling in CD4^+^ T cell immune responses, we generated mice in which Mib1 was conditionally inactivated under the control of the interferon-inducible promoter *Mx1* (*Mx1-Cre;Mib1^f/f^*) [30]. The Mib1-null DCs derived from the bone marrow (BM) of the *Mx1-Cre;Mib1^f/f^* mice failed to activate Notch signaling in Notch1-expressing C2C12 cells and in naïve CD4^+^ T cells. Nevertheless, the CD4^+^ T cells stimulated by Mib1-null DCs possessed comparable levels of the activation markers CD25, CD44, and CD69 and normal proliferation kinetics. Moreover, Th1 differentiation, which yields IFN-γ remained intact in the CD4^+^ T cells stimulated by Mib1-null DCs; this showed that Notch signaling in the context of DC-CD4^+^ T cell interaction is not required for these processes. In contrast, Th2 differentiation, which yields IL-4, was impaired dramatically both in vitro and in vivo. These data clearly indicate that Mib1-initiated Notch activation has a specific role in the promotion of Th2 differentiation.

## Materials and Methods

### Ethics Statement

All animal experiments were done with the approval of the ethical committees at the Seoul National University.

### Mice

The *Mib1^f/f^* mice were generated previously [23]. *Mx1-Cre* transgenic mice, OT-II TCR transgenic mice, and CD45.1 congenic mice were purchased from The Jackson Laboratory. For all experiments, we bred *Mx1-Cre;Mib1^f/f^* mice with *Mib1^f/f^* mice and examined the pups. To remove the floxed allele, 8-week-old *Mx1-Cre;Mib1^f/f^* and *Mib1^f/f^* mice received 4 i.p. injections of 300 µg of polyinosinic-polycytidylic acid (pIpC, Amersham Biosciences) at 2-day intervals. All the mouse lines were bred onto a C57BL/6 background (backcrossed more than 10 generations) and were maintained under specific pathogen-free conditions at the Seoul National University Animal Facility.

### Preparation of BM-derived DCs

BM cells were obtained from the tibias and femurs of mice 1 month after the last pIpC injection and 7.5×10^5^ cells were plated in non-culture treated 6-well plates in 2 ml of RPMI and 10% heat-inactivated FBS in the presence of 20 ng/ml of recombinant GM-CSF (Peprotech). On day 4, an equal amount of media containing rGM-CSF was added. On days 7 and 9, half of the media was replaced with fresh media. BM-derived DCs were harvested on day 9 and yielded 90∼95% CD11c^+^ cells. For DC stimulation, 1 µg/ml of LPS (Sigma-Aldrich) was added and incubated for 24 h.

### Preparation of naive CD4^+^ T cells

Lymph-node cells were collected from 8-week-old OT-II or SMARTA mice and incubated with biotin-conjugated Abs, anti-B220, CD8, CD11b, CD11c, CD19, CD25, CD69, Dx5, Gr-1, and Ter119 (all Abs were purchased from Biolegend). Untouched naive CD4^+^ T cells were negatively isolated by using streptavidin-coated magnetic beads (Invitrogen) or cell sorting with FACS Aria II (BD), which yielded greater than 95% purity.

### Isolation of DC precursors in BM and flow cytometry

BM precursors of classical spleen DC were isolated by FACS Aria II (BD), as reported previously [31]. Briefly, whole BM cells from 8-week-old C57BL/6 mice were stained with anti-CX3CR1-FITC, CD115-PE, CD135-APC, cKit-APC/Cy7, and Lin (CD3, CD19, NK1.1, Ter119, B220, CD11c, CD11b, and Gr1)-biotin Abs for MP (Lin^−^, Flt3^+^,cKit^hi^CX3CR1^−^), MDP (Lin^−^, Flt3^+^, cKit^+^, CX3CR1^+^), and CDP (Lin^−^, Flt3^+^, cKit^lo^, CD115^+^, CX3CR1^+^) or anti-SIRPα-FITC, I-A^b^-PE, CD135-APC, CD11c-APC/Cy7, and Lin2 (CD3, CD19, NK1.1, Ter119, and B220)-biotin Abs for pre-cDC (Lin2^−^, CD11c^+^, I-A^b−^, Flt3^+^, SIRPα^lo^). Streptavidin-PerCP was used as a secondary reagent. Rabbit polyclonal Abs raised against CX3CR1 (Abcam) were conjugated to FITC by using the EasyLink FITC conjugation Kit (Abcam). All other Abs were obtained from Biolegend, except for the PE-conjugated anti-Jag1 Ab (Lifespan biosciences).

### In vitro experiments

The LPS-stimulated DCs derived from *Mib1^f/f^* and *Mx1-Cre;Mib1^f/f^* mice were pretreated with 10 µg/ml of OVA_323–339_ or GP_61–80_ peptides for 6 h. Naïve CD4^+^ T cells (1×10^6^) were cultured with peptide-pretreated DCs (1×10^5^). After 5 days of incubation, cells were re-stimulated with 50 ng/ml of PMA (Sigma-Aldrich) and 0.5 µM ionomycin (Sigma-Aldrich) for 6 h in the presence of 2 µM monensin (Sigma-Aldrich). Cells were washed, fixed in 2% paraformaldehyde, and permeabilized in 0.5% saponin (Sigma-Aldrich). A neutralizing anti-IFN-γ Ab (XMG1.2, BD Pharmingen) was treated (10 µg/ml) for an experiment. For ELISA assays, viable T cells were harvested and equal numbers of cells per group were re-stimulated with 1 µg/ml plate-bound anti-CD3. The supernatants were removed after 48 h, and cytokine concentrations were determined by ELISA (OptEIA, BD). 5, 6-carboxyfluorescein diacetate succinimidyl ester (CFSE) labeling was performed as described previously [5]. For RT-PCR analyses, RNA was extracted from the negatively purified CD4^+^ T cells by using the RNeasy Micro kit (QIAGEN), according to the manufacturer's instructions. The isolated RNA was converted into cDNA by using Promega's RT system (Promega) with oligo-dT priming. The cDNAs from specific mRNA transcripts were quantified using quantitative real-time RT-PCR (Applied Biosystems) and SYBR Green technology (SYBR Premix Ex Taq, Takara). β-Actin was used as an internal control. The primer information will be provided on request. Protein extraction and western blot analyses were performed as described previously [18]. For the CBF-luciferase (luc) assay, the 8× WT or MT CBF-luc vectors were transfected into C2C12-Notch1 cells with pRL-TK vector using Lipofectamine (Invitrogen) as previously reported [26]. Luc activities were measured with a Dual Luciferase kit (Promega).

### Adoptive transfer experiment

1×10^6^ OT-II naive CD4^+^ T cells were intravenously transferred into CD45.1 recipient mice. After 24 h, the mice were intraperitoneally injected with 5×10^5^ peptide-preloaded DCs from *Mib1^f/f^* or *Mx1-Cre;Mib1^f/f^* mice. Seven days after the injection, splenocytes were harvested, resuspended at 1×10^7^/ml, and restimulated for 6 h with 10 µg/ml OVA_323–339_ in the presence of 50 U/ml human rIL-2 (Biolegend). Th cell populations in the OT-II CD4^+^ T cells (CD45.2^+^) and cytokine concentrations in the media were determined by flow cytometry and ELISA, respectively.

### Statistical analysis

All values are given as mean ± SD. Statistical comparisons were made by 2-tailed unpaired Student *t* test. A *P* value of less than 0.05 was considered statistically significant.

## Results

### Expression of ligands and E3 ligases in DCs for Notch signaling

**Figure 1:**
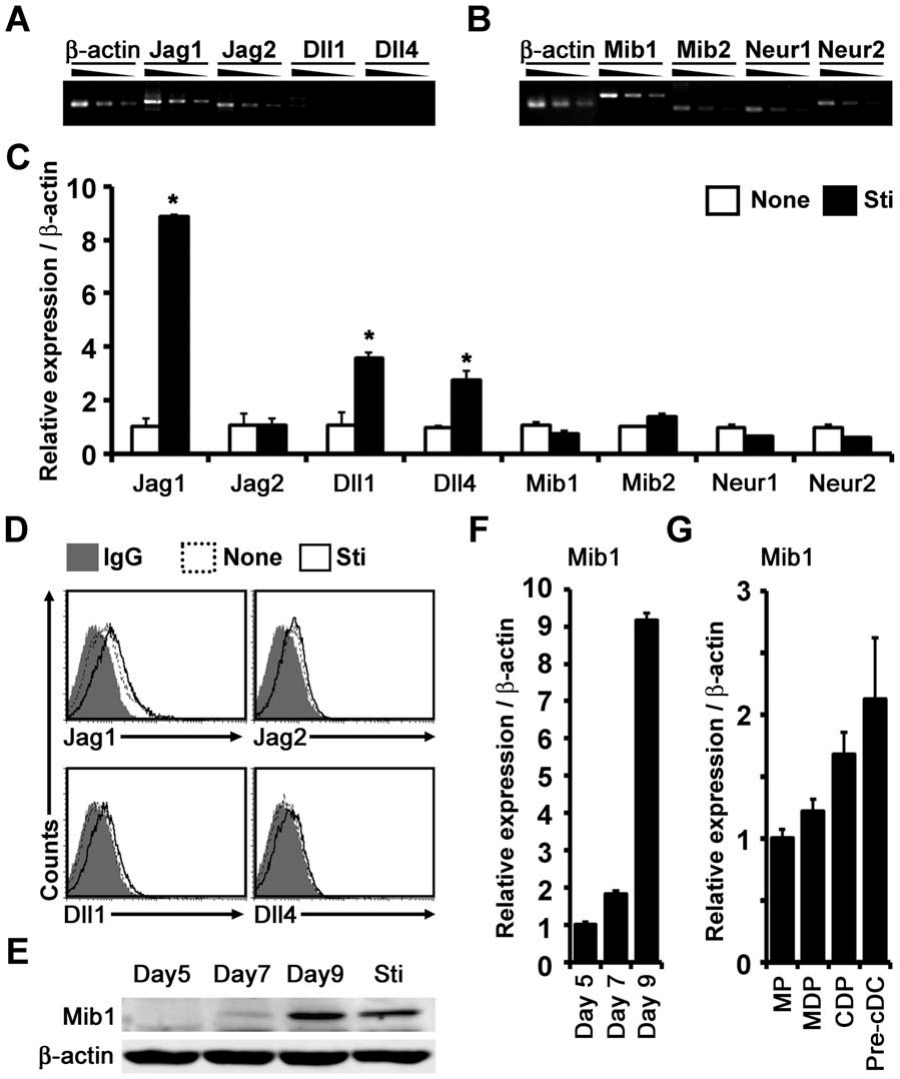
Expression of ligands and E3 ligases for Notch signaling in DCs. *A* and *B*, Semi-quantitative RT-PCR analyses of Notch ligands (*A*) and E3 ubiquitin ligases (*B*) in BM-derived DCs prior to LPS stimulation. Serial 2-fold dilutions of PCR products were electrophoresed in agarose gel *C*, Quantitative real-time RT-PCR analysis of ligands and E3 ligases in DCs before (*None*) and after 24 h of LPS stimulation (*Sti*). The results are representative of more than five independent experiments. *D*, Flow cytometry analysis for Notch ligands in DCs. IgG, Armenian hamster IgG isotype control; None, DCs before LPS stimulation; Sti, DCs after LPS stimulation. Results are representative of three independent experiments. *E*, Western blot analysis of Mib1 in BM culture with rGM-CSF on days 5, 7, and 9, and 24 h after LPS treatment on day9 (*Sti*). *F*, Quantitative real-time RT-PCR analysis of Mib1 in BM culture with rGM-CSF on days 5, 7, and 9. *G*, Quantitative real-time RT-PCR analysis of Mib1 in the BM precursors of classical spleen DC. MP, myeloid progenitor; MDP, macrophage and DC precursor; CDP, common DC precursor; pre-cDC, committed precursors of classical spleen DC. Results are representative of three independent experiments. Data represent mean ± SD; *, P<0.05.

First, we examined the expression of Notch ligands and their regulators, E3 ubiquitin ligases in BM-derived DCs. As reported previously [6], *Jag1* and *Jag2*, but not *Dll1* and *Dll4*, were substantially expressed (Fig. 1*A*). Among the four E3 ligases, *Mib1* was dominantly expressed, whereas *Mib2*, *Neur1*, and *Neur2* were detected at lower levels (Fig. 1*B*). Next, we investigated whether the expression of these E3 ligases are further induced by TLR stimulation. Although the transcripts and surface expression of Jag1, Dll1, and Dll4 increased in response to LPS treatment in accordance with a previous report [6], the expression profiles of *Mib1*, *Mib2*, *Neur1*, and *Neur2* were not influenced significantly (Fig. 1*C and D*), which suggests that the expressions of the Notch ligands and E3 ligases are regulated by distinct mechanisms.

Since among the E3 ligases, Mib1 is dominantly and constitutively expressed in BM-derived DCs, we investigated whether its expression is increased during BM-cell culture containing rGM-CSF. Interestingly, the Mib1 expression was increased dramatically according to the duration of culture (Fig. 1*E and F*), suggesting that Mib1 expression increases as the precursor cells differentiate into DCs. Consistent with mRNA transcripts, Mib1 protein expression was not induced any further in response to LPS (Fig. 1*E*). To confirm Mib1 expression during DC differentiation in vivo, we isolated DC precursors at various developmental stages from mice. In the mouse BM, myeloid progenitors (MPs) first differentiate into macrophage and DC precursors (MDPs), then into common DC precursors (CDPs), and finally into committed precursors of classical spleen DC (pre-cDCs) [31]. Interestingly, throughout this progression, the relative Mib1 transcript levels increased progressively (Fig. 1*G*). Taken together, these results show that Mib1 expression in DCs increases with progress in the development, suggesting a potential role of Mib1 in peripheral DCs.

### Mib1-null DCs show normal development and Notch ligands expression

**Figure 2:**
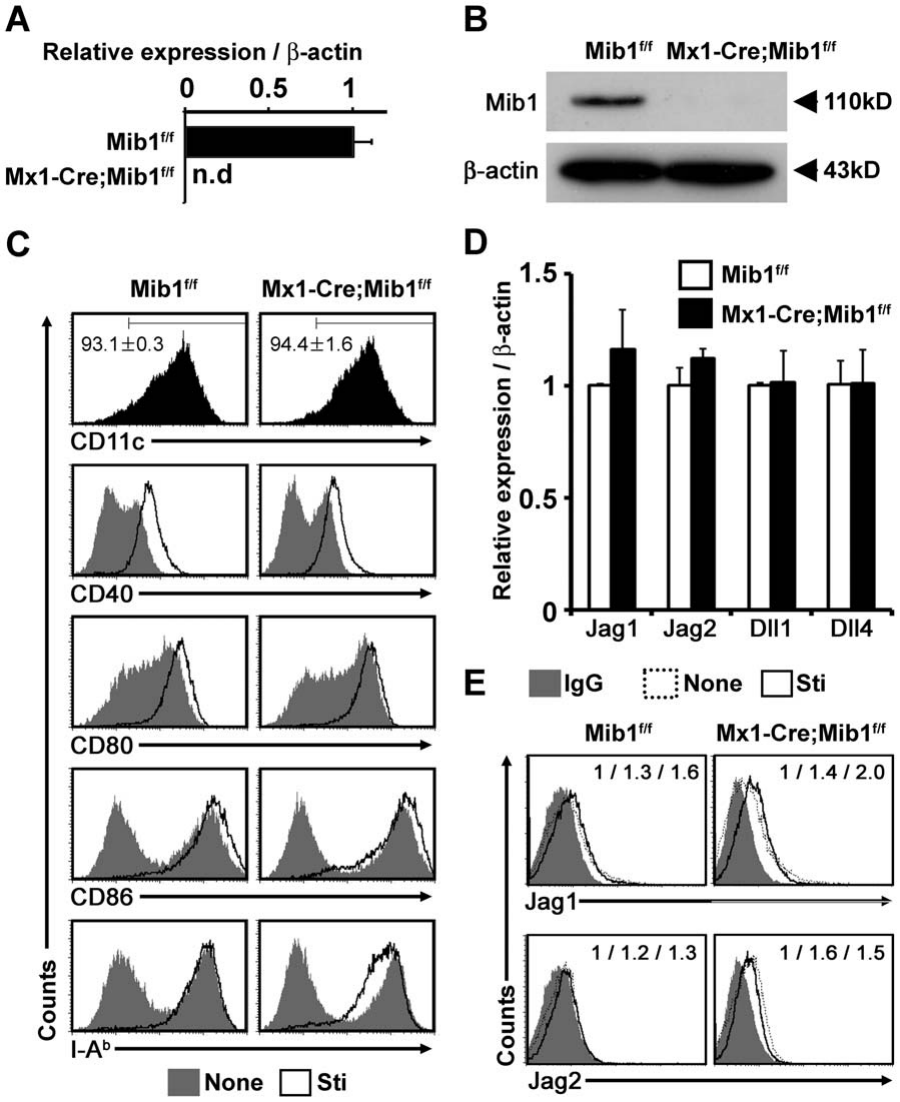
Generation of Mib1-null DCs. *A* and *B*, Quantitative real-time RT-PCR (*A*) and western blot (*B*) analyses of Mib1 in DCs from *Mib1^f/f^* or *Mx1-Cre;Mib1^f/f^* mice. *C*, Flow cytometry analysis for DC marker, CD11c, costimulatory molecules, CD40, CD80 (B7.1), and CD86 (B7.2), and MHC II (I-A^b^) in *Mib1^f/f^* or *Mx1-Cre;Mib1^f/f^* DCs before (*None, gray-filled histogram*) or after LPS stimulation (*Sti, black line*). Numbers on flow cytometry plots represent the mean percentage of cells ± SD. *D*, Quantitative real-time RT-PCR analysis of Notch ligands in LPS-stimulated DCs from *Mib1^f/f^* and *Mx1-Cre;Mib1^f/f^* mice. Results are representative of three independent experiments. *E*, Flow cytometry analysis for Jag1 and Jag2 in *Mib1^f/f^* or *Mx1-Cre;Mib1^f/f^* DCs. Numbers indicate the relative median fluorescence intensities of IgG isotype control (*IgG, gray-filled histogram*), DCs before LPS stimulation (*None, dotted line*), and DCs after LPS stimulation (*Sti, black line*), respectively, in each plots. Data represent mean ± SD.

To investigate the role of Mib1 in DC-mediated immune responses, we inactivated Mib1 in hematopoietic systems, including hematopoietic stem cells, by crossing Mib1-floxed mice (*Mib1^f/f^*) with *Mx1-Cre* transgenic mice; this enabled the expression of Cre recombinase in response to pIpC [23], [25], [30]. The mRNA and protein expression of Mib1 disappeared in the BM-derived DCs that originated from *Mx1-Cre;Mib1^f/f^* mice (Fig. 2*A and B*). Although several studies have implicated Notch signaling at various stages of DC generation and maturation [3], [32], Mib1-null DCs were derived from BM hematopoietic precursors easily and displayed comparable levels of the maturation markers CD40, CD80, CD86, and MHC-II, both before and after LPS stimulation (Fig. 2*C*). This results show that Mib1 is dispensable for DC development in a cell-autonomous manner.

Next, we examined the expression of Notch ligands in Mib1-null DCs. The transcript levels for Notch ligands in Mib1-null DCs were comparable to those in control (*Mib1^f/f^*) DCs (Fig. 2*D*). However, Mib1-null DCs showed accumulation of surface Jag1 and Jag2 proteins (Fig. 2*E*); this finding is consistent with those of previous studies, which reported that impaired endocytosis of Notch ligands resulted in the accumulation of Notch ligands on the cell surface [25], [33], [34]. These data showed that *Mx1-Cre;Mib1^f/f^* DCs, in which Mib1 is completely deleted, exhibited intact DC phenotypes including Notch ligands expression.

### Mib1-null DCs cannot trigger Notch signaling in adjacent cells

**Figure 3:**
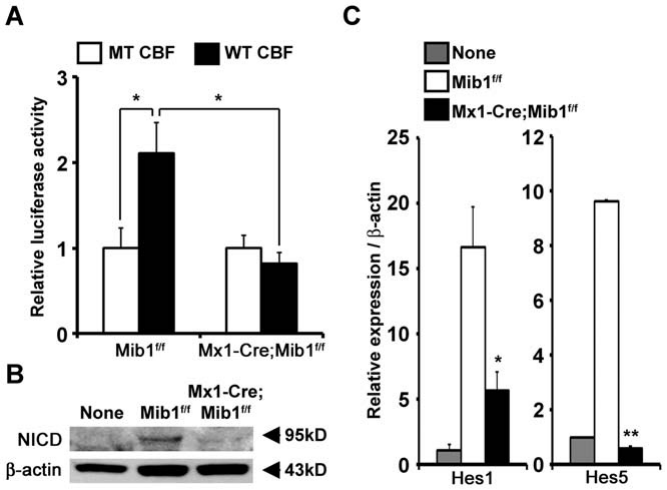
Mib1-null DCs fail to activate Notch signaling. A, LPS-stimulated *Mib1^f/f^* or *Mx1-Cre;Mib1^f/f^* DCs were cultured with Notch1-expressing C2C12 cells transfected with WT or MT CBF luciferase constructs, and 24 h after the coculture, luciferase activity was measured. Results are representative of two independent experiments. *B*, Western blot analysis of Notch1 ICD (NICD) in negatively purified OT-II CD4^+^ T cells before (*None*) and 6 h after coculture with peptide-pretreated *Mib1^f/f^* or *Mx1-Cre;Mib1^f/f^* DCs. *C*, Quantitative real-time RT-PCR analysis of Notch target genes, Hes1 and Hes5, in CD4^+^ T cells before (*None*) and after coculture with *Mib1^f/f^* or *Mx1-Cre;Mib1^f/f^* DCs. Results are representative of three independent experiments. Data represent mean ± SD; *, P<0.05; **, P<0.001.

To determine whether Mib1-null DCs were capable of triggering the activation of Notch signaling in the adjacent cells, we transfected Notch1-expressing C2C12 cells with WT and MT CBF-luc constructs [26] and co-cultured the transfected cells with control and Mib1-null DCs. Notably, Mib1-null DCs failed to induce CBF-luc activity (Fig. 3*A*). Next, we co-cultured purified OT-II naive CD4^+^ T cells with OVA_323–339_ peptide-loaded control and Mib1-null DCs. As a result, we observed strikingly increased NICD in the CD4^+^ T cells cultured with control DCs, but not in the CD4^+^ T cells cultured with Mib1-null DCs (Fig. 3*B*). Moreover, the expressions of Notch target genes, Hes1 and Hes5, in CD4^+^ T cells were not markedly induced by Mib1-null DCs (Fig. 3*C*). Collectively, ablation of Mib1 in DCs led to incapability of Notch signaling activation, in spite of normal or even elevated levels of Notch ligands.

### Mib1-null DCs readily induce the activation and proliferation of CD4^+^ T cells

**Figure 4:**
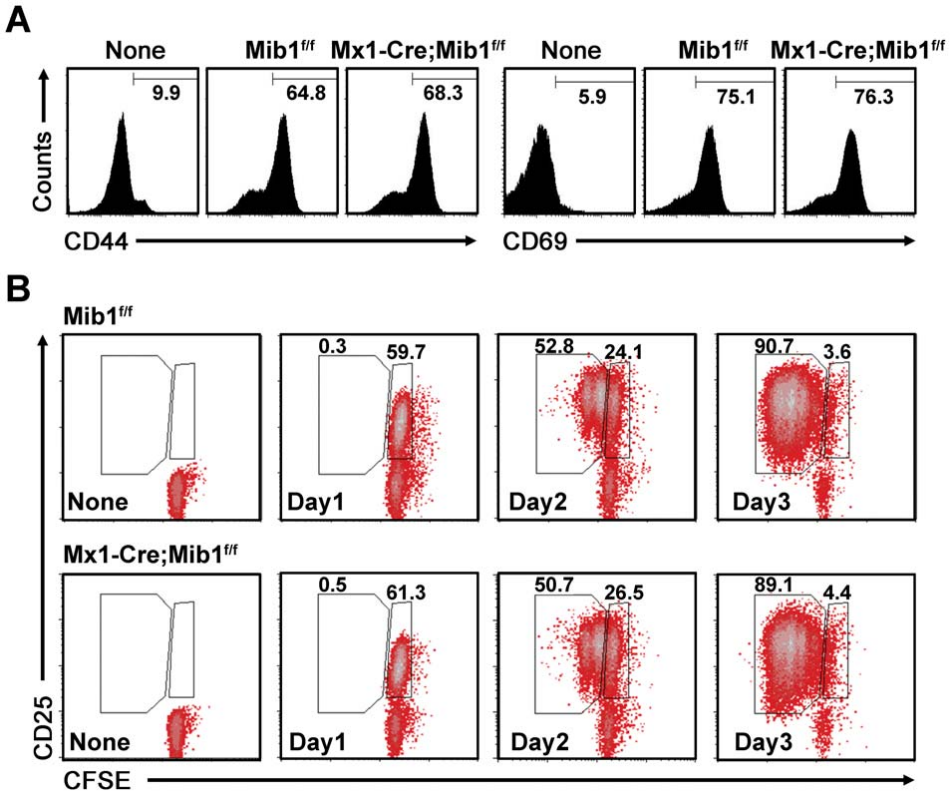
Preserved activation and proliferation of Notch-inactivated CD4^+^ T cells. *A*, Flow cytometry analysis for T cell activation markers, CD44 and CD69, in purified CD4^+^ T cells before (None) or 24 h after coculture with *Mib1^f/f^* or *Mx1-Cre;Mib1^f/f^* DCs. *B*, CFSE-labeled naive OT-II CD4^+^ T cells were cultured without (*None*) or with peptide-pretreated *Mib1^f/f^* or *Mx1-Cre;Mib1^f/f^* DCs, and the activated cells collected on days 1, 2, and 3, respectively, were stained for CD25 and CD4 and analyzed by flow cytometry. The numbers indicate the percentage of cells within the gates. A representative of three independent experiments is shown.

To examine whether Mib1 ablation in DCs influence CD4^+^ T cell activation and proliferation, we further analyzed OT-II CD4^+^ T cells cultured with control or Mib1-null DCs. Intriguingly, however, Mib1-null DCs readily induced upregulation of T-cell activation markers CD25, CD44, and CD69, similar to the control DCs (Fig. 4 *A* and *B*). Moreover, the proliferation kinetics of CD4^+^ T cells cultured with the control or Mib1-null DCs were also comparable, as shown by CFSE dilution (Fig. 4 *B*). This suggested that CD4^+^ T cells can be readily activated and proliferate in the absence of Notch activation via interaction with DCs.

### Mib1-null DCs impair *in vitro* Th2 differentiation

**Figure 5:**
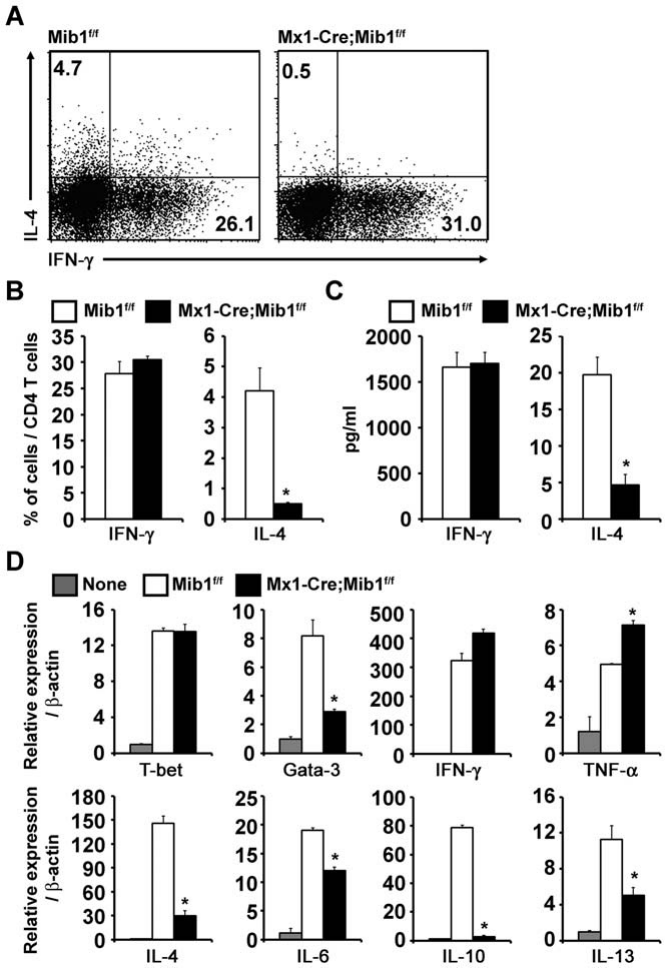
Absence of Mib1 in DCs specifically impairs Th2 differentiation. *A*, Purified naive OT-II CD4^+^ T cells were cultured with peptide pretreated *Mib1^f/f^* or *Mx1-Cre;Mib1^f/f^* DCs. After 5 days, viable cells were re-stimulated with PMA and ionomycin for 6 h, and the intracellular levels of IFN-γ and IL-4 were analyzed by flow cytometry. The numbers indicate the percentage of cells within the gates. A representative of three independent experiments is shown. *B*, The average percentage of activated CD4^+^ T cells producing IFN-γ and IL-4 (as in [*A*]) from three independent experiments. *C*, Viable activated CD4^+^ T cells were harvested, and an equal number of cells in each group were re-stimulated with 1 µg/ml plate-bound anti-CD3. The supernatants were removed after 48 h, and cytokine concentrations were determined by ELISA. *D*, Quantitative real-time RT-PCR analysis of T-bet, Gata-3, Th1 related cytokines (IFN-γ and TNF-α), and Th2 related cytokines (IL-4, IL-6, IL-10, and IL-13) in purified CD4^+^ T cells unstimulated (*None*) or stimulated by *Mib1^f/f^* or *Mx1-Cre;Mib1^f/f^* DCs. Data represent mean ± SD; *, P<0.05.

To investigate whether Mib1-null DCs are able to induce Th1/2 differentiation, we performed intracellular cytokine staining for IFN-γ and IL-4 in OT-II CD4^+^ T cells cultured with DCs. As a result, the percentage of IFN-γ-expressing cells was comparable between the control and Mib1-null DCs; however, the percentage of IL-4-expressing cells was markedly decreased in Mib1-null DCs (Fig. 5*A and B*). The CD4^+^ T cells stimulated by Mib1-null DCs also exhibited decreased IL-4 production when subjected to ELISA analysis (Fig. 5*C*). In addition, expressions of Gata-3, a central factor for the differentiation of Th2 cells, and various Th2 cytokines such as IL-4, IL-6, IL-10, and IL-13 were notably reduced in the CD4^+^ T cells cultured with Mib1-null DCs (Fig. 5*D*). In contrast, expressions of T-bet, a master regulator of Th1 differentiation, and Th1 cytokines including IFN-γ and TNF-α were comparable between the CD4^+^ T cells stimulated by the control and Mib1-null DCs (Fig. 5*D*).

**Figure 6:**
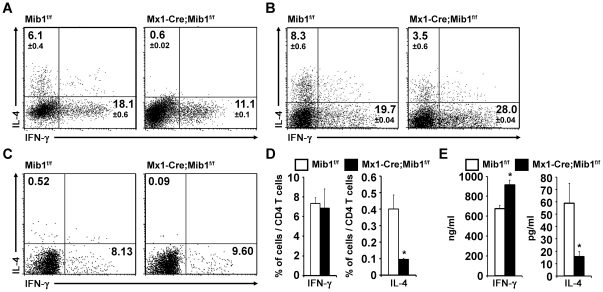
Mib1 in DCs is critical for Th2 induction. *A*, Neutralizing anti-IFN-γ Abs (10 µg/ml) were treated to CD4^+^ T cells during the coculture with peptide pretreated *Mib1^f/f^* or *Mx1-Cre;Mib1^f/f^* DCs. Intracellular cytokine expression was measured by flow cytometry, as shown in Figure 5*A*. The numbers indicate the mean ± SD cell percentages from two independent experiments. *B*, Purified naïve SMARTA CD4^+^ T cells were stimulated with peptide-pretreated *Mib1^f/f^* or *Mx1-Cre;Mib1^f/f^* DCs. Intracellular cytokine expression was measured by flow cytometry, as shown in Figure 5*A*. The numbers indicate the mean ± SD cell percentages from two independent experiments. *C*, Purified naive OT-II CD4^+^ T cells were transferred intravenously into CD45.1 recipient mice. Peptide-pretreated *Mib1^f/f^* or *Mx1-Cre;Mib1^f/f^* DCs were intraperitoneally injected on the subsequent day. Seven days later, splenocytes were re-stimulated for 6 h with OVA_323–339_ peptides and human rIL-2, and intracellular IFN-γ and IL-4 were analyzed from CD45.2^+^ CD4^+^ cells by flow cytometry. The numbers indicate the percentage of cells within the gates. A representative of three independent experiments is shown. *D*, The average percentage of activated IFN-γ and IL-4-producing CD45.2^+^ CD4^+^ T cells (as shown in [*C*]) from three independent experiments. *E*, IFN-γ and IL-4 productions were detected by ELISA 72 h after re-stimulation. *, P<0.05.

Next, we asked whether the suppression of Th2 differentiation by Mib1 ablation in DCs is due to an enhanced expression of IFN-γ [35], [36]. We treated neutralizing anti-IFN-γ Abs during the CD4^+^ T cells and DCs coculture, but the neutralization of IFN-γ could not rescued the impaired Th2 differentiation in CD4^+^ T cells stimulated by Mib1-null DCs (Fig. 6*A*), ruling out the possibility of the secondary effect of Notch signaling for the regulation of Th2 differentiation. To examine the role of Mib1 in Th differentiation further, we used another TCR-transgenic mouse model, SMARTA, which expresses a specific TCR for the lymphocytic choriomeningitis virus epitope GP_61–80_ [37]. As shown in Fig. 6B, the frequency of IL-4-expressing cells was lower in CD4^+^ T cells stimulated with Mib1-null DCs than in the control CD4^+^ T cells, implying that defective Th2 differentiation is not limited to certain TCR transgenic mice. LPS-stimulated DCs have been known to promote both Th1 and Th2 responses by expressing Dll and Jag, respectively [6], [38]. Unexpectedly, however, no defects in Th1 differentiation were observed, suggesting that Th1 differentiation does not require DC-mediated Notch activation. Overall, we showed that Mib1 deletion in DCs impaired Th2 differentiation by inactivating Notch signaling in CD4^+^ T cells.

### Mib1 in DCs is critical for Th2 induction *in vivo*

Finally, we used an adoptive transfer strategy to perform an *in vivo* experiment by transferring naïve OT-II CD4^+^ T cells and the control or Mib1-null DCs into CD45.1 congenic mice. Once again, the specific defect in Th2 differentiation in Mib1-null DCs was identified, while simultaneously preserving the Th1 responses, by using intracellular cytokine staining (Fig. 6*C and D*) and ELISA analyses (Fig. 6*E*) to measure IFN-γ and IL-4 expressions. In conclusion, the Mib1 expressed in DCs is a critical regulator of Notch activation during peripheral immune responses. The complete inactivation of Notch signaling in DC-CD4^+^ T cell interactions leads to dramatically impaired Th2 differentiation both *in vitro* and *in vivo*. However, there was no evidence of a direct association between Notch signaling and CD4^+^ T cell activation, proliferation, and Th1 differentiation.

## Discussion

Regulation of CD4^+^ T cell-mediated immune responses is crucial for protection against different types of pathogenic microorganisms as well as peripheral immune tolerance. Especially, a delicate balance of diverse Th subsets, Th1, Th2, Th17, and induced regulatory T cells, enables an effective elimination of dangerous microbes [39]. Although Notch signaling has been known as one of the mechanisms to regulate Th responses, the ambiguous role of Notch signaling has hampered any further clinical application. Previous studies have investigated the role of Notch signaling by using various experimental approaches, including overexpression of Notch ligands or NICD, ectopic manipulation of cytokines, pharmacological inhibition of Notch signaling by nonselective γ-secretase inhibitors, and treatment with agonistic or antagonistic antibodies [5], [7], [8], [9], [10], [11], [12]. Although these approaches are convenient and provide many important cues to reveal the role of Notch signaling, they could result in non-physiological levels of signaling and unknown side effects that need to be evaluated using genetic approaches.

The Mib1-null DCs used in this study served as a novel and reliable genetic model to modulate Notch signaling in CD4^+^ T cells for several reasons. First, Mib1 is dominantly expressed in DCs and known to play an obligatory role in regulating all Notch ligands in mice [23]. Indeed, Mib1-null DCs could not activate Notch signaling in adjacent cells, including CD4^+^ T cells, in spite of the Notch ligands expressions. Thus, the manipulation of Mib1 in DCs efficiently and effectively blocks Notch activation in CD4^+^ T cells. Second, using intact naive CD4^+^ T cells in both control and experimental groups, we could exclude the side effects caused by direct manipulation of CD4^+^ T cells and assure their potential capacity of activation and subset differentiation. Finally yet importantly, our approach is relevant to the physiological conditions for CD4^+^ T cell immune responses because the Mib1-null DCs can act as functional APCs, although they could not activate Notch signaling in the adjacent cells.

In this study, we clearly showed that Mib1 deficiency in DCs strengthen the Th2-regulating role rather than Th1 for Notch signaling. In addition, there was no evidence of a regulatory role for Notch signaling in CD4^+^ T cell activation and proliferation, which is consistent with other studies using independent genetic approaches, such as RBP-Jκ KO [6], Notch1 and Notch2 double KO [40], and transgenic overexpression of dominant negative MAML [14]. In a previous study, different Notch ligands, Dll and Jag on APCs induced Th1 and Th2, respectively, *in vitro* [6]. However, there is no evidence for ligand type-dependent regulation of Th differentiation in our approaches, because neither Jag nor Dll triggered Notch activation in the absence of Mib1.

The modulation of genes related to Notch signaling in APCs, the signal-sending cells, has not been understood well relative to that in effector T cells, the signal-receiving cells. We identified that Mib1 is dominantly expressed in DCs among four Notch-regulating E3 ligases, and Notch signaling is completely inactivated solely by Mib1 ablation. Unlike the Notch ligands, interestingly, Mib1 was highly expressed in DCs prior to antigen stimulation, enabling DCs to be in ‘ready to go’ state to activate Notch signaling. However, the molecular mechanism that regulates Mib1 expression during DC development as well as ligands expression after LPS stimulation has not yet been revealed.

Although recent studies have implicated Notch signaling in the differentiation and function of other Th subsets, such as Th17 [41] and induced regulatory T cells [42], [43], these roles of Notch signaling have not been evaluated clearly by using genetic methods. Further studies are required to reveal the role of Notch signaling in different types of peripheral immune responses other than Th1/Th2 differentiation. Here again we expect that our approach can be applicable to those expanded issues, and even more reliable than others because we can assure the potential capacity of Th subset differentiation. Furthermore, our results suggest that Mib1 is a latent therapeutic target for the regulation of Notch activation in disorders caused by a predominance of Th2 cell-cytokines, such as allergies, asthma, atopic dermatitis, systemic lupus erythematosus, and chronic graft-versus-host disease.

## References

[1] Amsen D, Antov A, Flavell RA (2009). The different faces of Notch in T-helper-cell differentiation.. Nat Rev Immunol.

[2] Osborne BA, Minter LM (2007). Notch signalling during peripheral T-cell activation and differentiation.. Nat Rev Immunol.

[3] Yuan JS, Kousis PC, Suliman S, Visan I, Guidos CJ (2009). Functions of notch signaling in the immune system: consensus and controversies.. Annu Rev Immunol.

[4] Kopan R, Ilagan MX (2009). The canonical Notch signaling pathway: unfolding the activation mechanism.. Cell.

[5] Adler SH, Chiffoleau E, Xu L, Dalton NM, Burg JM (2003). Notch signaling augments T cell responsiveness by enhancing CD25 expression.. J Immunol.

[6] Amsen D, Blander JM, Lee GR, Tanigaki K, Honjo T (2004). Instruction of distinct CD4 T helper cell fates by different notch ligands on antigen-presenting cells.. Cell.

[7] Maekawa Y, Tsukumo S, Chiba S, Hirai H, Hayashi Y (2003). Delta1-Notch3 interactions bias the functional differentiation of activated CD4+ T cells.. Immunity.

[8] Palaga T, Miele L, Golde TE, Osborne BA (2003). TCR-mediated Notch signaling regulates proliferation and IFN-gamma production in peripheral T cells.. J Immunol.

[9] Eagar TN, Tang Q, Wolfe M, He Y, Pear WS (2004). Notch 1 signaling regulates peripheral T cell activation.. Immunity.

[10] Kostianovsky AM, Maier LM, Baecher-Allan C, Anderson AC, Anderson DE (2007). Up-regulation of gene related to anergy in lymphocytes is associated with Notch-mediated human T cell suppression.. J Immunol.

[11] Minter LM, Turley DM, Das P, Shin HM, Joshi I (2005). Inhibitors of gamma-secretase block in vivo and in vitro T helper type 1 polarization by preventing Notch upregulation of Tbx21.. Nat Immunol.

[12] Sun J, Krawczyk CJ, Pearce EJ (2008). Suppression of Th2 cell development by Notch ligands Delta1 and Delta4.. J Immunol.

[13] Tanigaki K, Tsuji M, Yamamoto N, Han H, Tsukada J (2004). Regulation of alphabeta/gammadelta T cell lineage commitment and peripheral T cell responses by Notch/RBP-J signaling.. Immunity.

[14] Tu L, Fang TC, Artis D, Shestova O, Pross SE (2005). Notch signaling is an important regulator of type 2 immunity.. J Exp Med.

[15] Ong CT, Sedy JR, Murphy KM, Kopan R (2008). Notch and presenilin regulate cellular expansion and cytokine secretion but cannot instruct Th1/Th2 fate acquisition.. PLoS One.

[16] Tacchini-Cottier F, Allenbach C, Otten LA, Radtke F (2004). Notch1 expression on T cells is not required for CD4+ T helper differentiation.. Eur J Immunol.

[17] Weinmaster G, Fischer JA (2011). Notch ligand ubiquitylation: what is it good for?. Dev Cell.

[18] Koo BK, Lim HS, Song R, Yoon MJ, Yoon KJ (2005). Mind bomb 1 is essential for generating functional Notch ligands to activate Notch.. Development.

[19] Koo BK, Yoon KJ, Yoo KW, Lim HS, Song R (2005). Mind bomb-2 is an E3 ligase for Notch ligand.. J Biol Chem.

[20] Ruan Y, Tecott L, Jiang MM, Jan LY, Jan YN (2001). Ethanol hypersensitivity and olfactory discrimination defect in mice lacking a homolog of Drosophila neuralized.. Proc Natl Acad Sci U S A.

[21] Song R, Koo BK, Yoon KJ, Yoon MJ, Yoo KW (2006). Neuralized-2 regulates a Notch ligand in cooperation with Mind bomb-1.. J Biol Chem.

[22] Vollrath B, Pudney J, Asa S, Leder P, Fitzgerald K (2001). Isolation of a murine homologue of the Drosophila neuralized gene, a gene required for axonemal integrity in spermatozoa and terminal maturation of the mammary gland.. Mol Cell Biol.

[23] Koo BK, Yoon MJ, Yoon KJ, Im SK, Kim YY (2007). An obligatory role of mind bomb-1 in notch signaling of mammalian development.. PLoS One.

[24] Kim YW, Koo BK, Jeong HW, Yoon MJ, Song R (2008). Defective Notch activation in microenvironment leads to myeloproliferative disease.. Blood.

[25] Song R, Kim YW, Koo BK, Jeong HW, Yoon MJ (2008). Mind bomb 1 in the lymphopoietic niches is essential for T and marginal zone B cell development.. J Exp Med.

[26] Yoon MJ, Koo BK, Song R, Jeong HW, Shin J (2008). Mind bomb-1 is essential for intraembryonic hematopoiesis in the aortic endothelium and the subaortic patches.. Mol Cell Biol.

[27] Jeong HW, Jeon US, Koo BK, Kim WY, Im SK (2009). Inactivation of Notch signaling in the renal collecting duct causes nephrogenic diabetes insipidus in mice.. J Clin Invest.

[28] Koo BK, Lim HS, Chang HJ, Yoon MJ, Choi Y (2009). Notch signaling promotes the generation of EphrinB1-positive intestinal epithelial cells.. Gastroenterology.

[29] Yoon KJ, Koo BK, Im SK, Jeong HW, Ghim J (2008). Mind bomb 1-expressing intermediate progenitors generate notch signaling to maintain radial glial cells.. Neuron.

[30] Kuhn R, Schwenk F, Aguet M, Rajewsky K (1995). Inducible gene targeting in mice.. Science.

[31] Liu K, Victora GD, Schwickert TA, Guermonprez P, Meredith MM (2009). In vivo analysis of dendritic cell development and homeostasis.. Science.

[32] Zhou J, Cheng P, Youn JI, Cotter MJ, Gabrilovich DI (2009). Notch and wingless signaling cooperate in regulation of dendritic cell differentiation.. Immunity.

[33] De Renzis S, Yu J, Zinzen R, Wieschaus E (2006). Dorsal-ventral pattern of Delta trafficking is established by a Snail-Tom-Neuralized pathway.. Dev Cell.

[34] Itoh M, Kim CH, Palardy G, Oda T, Jiang YJ (2003). Mind bomb is a ubiquitin ligase that is essential for efficient activation of Notch signaling by Delta.. Dev Cell.

[35] Maggi E, Parronchi P, Manetti R, Simonelli C, Piccinni MP (1992). Reciprocal regulatory effects of IFN-gamma and IL-4 on the in vitro development of human Th1 and Th2 clones.. J Immunol.

[36] Scott P (1991). IFN-gamma modulates the early development of Th1 and Th2 responses in a murine model of cutaneous leishmaniasis.. J Immunol.

[37] Oxenius A, Bachmann MF, Zinkernagel RM, Hengartner H (1998). Virus-specific MHC-class II-restricted TCR-transgenic mice: effects on humoral and cellular immune responses after viral infection.. Eur J Immunol.

[38] Eisenbarth SC, Piggott DA, Huleatt JW, Visintin I, Herrick CA (2002). Lipopolysaccharide-enhanced, toll-like receptor 4-dependent T helper cell type 2 responses to inhaled antigen.. J Exp Med.

[39] Zhu J, Yamane H, Paul WE (2010). Differentiation of effector CD4 T cell populations.. Annu Rev Immunol.

[40] Amsen D, Antov A, Jankovic D, Sher A, Radtke F (2007). Direct regulation of Gata3 expression determines the T helper differentiation potential of Notch.. Immunity.

[41] Ito T, Schaller M, Hogaboam CM, Standiford TJ, Sandor M (2009). TLR9 regulates the mycobacteria-elicited pulmonary granulomatous immune response in mice through DC-derived Notch ligand delta-like 4.. J Clin Invest.

[42] Samon JB, Champhekar A, Minter LM, Telfer JC, Miele L (2008). Notch1 and TGFbeta1 cooperatively regulate Foxp3 expression and the maintenance of peripheral regulatory T cells.. Blood.

[43] Ostroukhova M, Qi Z, Oriss TB, Dixon-McCarthy B, Ray P (2006). Treg-mediated immunosuppression involves activation of the Notch-HES1 axis by membrane-bound TGF-beta.. J Clin Invest.

