# Correction: Mind Bomb-1 in Dendritic Cells Is Specifically Required for Notch-mediated T Helper Type 2 Differentiation

**DOI:** 10.1371/annotation/14bcb316-f12f-45ae-a1d3-d71a6f2014ce

**Published:** 2012-07-12

**Authors:** Hyun-Woo Jeong, Ji-Hoon Kim, Joo-Yeon Kim, Sang-Jun Ha, Young-Yun Kong

Information is missing from the Funding statement. The complete Funding statement should read: "This work was supported by grants from the Basic Science Research Program through the National Research Foundation of Korea (2012-0000121); the Bio & Medical Technology Development Program of the National Research Foundation (NRF) funded by the Korean government (MEST) (2011-0019269); National R&D Program for Cancer Control, Ministry of Health & Welfare, the Republic of Korea (0920310); and the National Research Foundation of Korea Grant funded by the Korean Government (MEST) (NRF-M1AXA002-2011-0028413 and NRF-2011-355-C00079). The funders had no role in study design, data collection and analysis, decision to publish, or preparation of the manuscript."

